# Conservation physiology of freshwater fishes: an illustration of pressing questions and implications for management

**DOI:** 10.1093/conphys/coaf057

**Published:** 2025-08-04

**Authors:** Naomi Pleizier, Gail D Schwieterman, Kim Birnie-Gauvin, Tamzin A Blewett, Terra L Dressler, Emily A Hardison, Ken M Jeffries, Krista Kraskura, Amy K Teffer, Jacey C Van Wert, Erika J Eliason

**Affiliations:** Department of Ecology, Evolution and Marine Biology, University of California Santa Barbara, Santa Barbara CA 93106 USA; Department of Ecology, Evolution and Marine Biology, University of California Santa Barbara, Santa Barbara CA 93106 USA; School of Marine Sciences, University of Maine, Orono, ME 04469 USA; Maine Agricultural and Forest Experiment Station, University of Maine, Orono, ME 04469 USA; Department of Ecology, Evolution and Marine Biology, University of California Santa Barbara, Santa Barbara CA 93106 USA; Section for Freshwater Fisheries and Ecology, Technical University of Denmark, Silkeborg, Denmark; Department of Biological Sciences, University of Alberta, Edmonton, Alberta T6G 2R3, Canada; Department of Ecology, Evolution and Marine Biology, University of California Santa Barbara, Santa Barbara CA 93106 USA; Stillwater Sciences, 996 S. Seaward Avenue, Suite 102, Ventura, CA 93001, USA; Department of Ecology, Evolution and Marine Biology, University of California Santa Barbara, Santa Barbara CA 93106 USA; Department of Biological Sciences, University of Pittsburgh, Pittsburgh, PA 15206, USA; Department of Biological Sciences, University of Manitoba, Winnipeg, Manitoba R3T 2N2, Canada; Department of Ecology, Evolution and Marine Biology, University of California Santa Barbara, Santa Barbara CA 93106 USA; Department of Biological Sciences, Towson University, Towson, MD 21252 USA; Department of Environmental Conservation, University of Massachusetts Amherst, Amherst MA 01003, USA; Department of Ecology, Evolution and Marine Biology, University of California Santa Barbara, Santa Barbara CA 93106 USA; UF/IFAS SFFGS Fisheries and Aquatic Sciences Program, University of Florida, Gainesville, FL 32653, USA; Department of Ecology, Evolution and Marine Biology, University of California Santa Barbara, Santa Barbara CA 93106 USA; Fisheries and Oceans Canada, Pacific Science Enterprise Center, Fisheries and Oceans Canada, West Vancouver, BC V7V 1H2, Canada

**Keywords:** Climate Change, Contaminants, Eutrophication, Food Availability, Habitat Fragmentation, Infectious Disease, Invasive Species, Light Pollution, Natural Disasters, Sound Pollution

## Abstract

Rivers, lakes, and wetlands are facing threats that continue to grow in intensity and frequency from climate change, habitat fragmentation, invasive species, changes in food availability, natural disasters, various forms of pollution (*e.g.,* trace metals, light, noise), and emerging infectious diseases. These disruptions to freshwater environments are driving population declines in freshwater fishes as well as threatening migratory species that need freshwater habitats to complete their life cycle. To improve freshwater fish conservation efforts, it is essential to understand the magnitude and nature of the threats fish are currently facing. Here, we present a series of case studies that illustrate the utility of employing physiological methods to assess both the threats facing freshwater fishes, and the conservation efforts being used to help preserve freshwater biodiversity. We present an array of physiological tools that can be used across multiple levels of biological organization, from molecular to population-level, to address a variety of questions. Finally, we share what we view to be pressing questions in freshwater fish conservation physiology and highlight strategies to help bridge gaps across different user groups.

## Abbreviations

ABT, Arrhenius Breakpoint Temperature
Ag^+^, Silver
ALAN, Artificial light at night
AS, Aerobic Scope
ATP, Adenosine Triphosphate
CCME, Canadian Council of Ministers of the Environment
Cl^-^, Chloride
CT_max_^,^ Critical Thermal Maximum
MMR, Maximum Metabolic Rate
NO_2_^-^, Nitrites
NO_3_^-^, Nitrates
PIT, Passive Integrated Transponder
SMR, Standard Metabolic Rate
TFM, 3-trifluoromethyl-4-nitrophenol
T_opt,_ Optimal Temperatures for Performance

## Introduction

Freshwater ecosystems are home to more than 18000 species of fish (approximately 51% of known fish species; Strayer and Dudgeon, 2010); but represent a mere 1% of global aquatic ecosystems. Fresh waters are therefore biodiversity hotspots, though they tend to be viewed as a resource to be exploited, rather than a diverse habitat in need of protection (Birnie-Gauvin *et al.*, 2023). Freshwater fishes have declined by an astounding 76% since 1970, with the abundance of freshwater mega-fishes (>30 kg) having declined by 94% (Deinet *et al.*, 2020). Rivers, lakes, and wetlands are facing threats that continue to grow in intensity and frequency, including climate change, habitat fragmentation, infectious diseases, invasive species, various forms of pollution (*e.g.,* microplastics, sewage, light, noise), and many others (Dudgeon *et al.*, 2006, Reid *et al.*, 2019). These disruptions to freshwater environments are driving population declines in freshwater species as well as threatening migratory species that need both saltwater and freshwater habitats to complete their life cycle. Whereas some threats to fishes are common across the globe (*e.g.,* climate change, overfishing), freshwater systems face unique issues that hinder conservation and sustainable management efforts.

**Figure 1:**
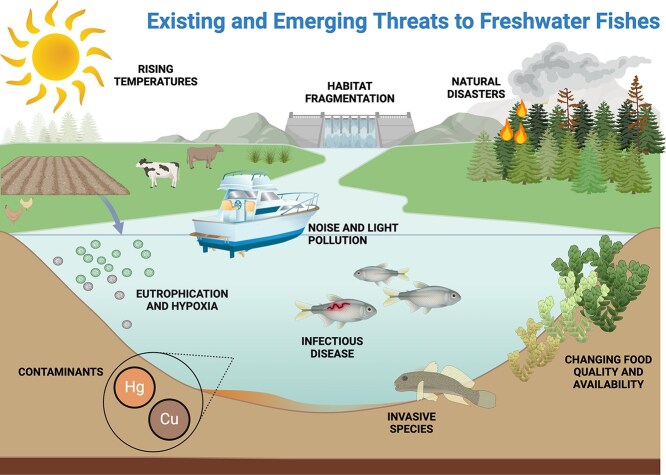
Diverse threats facing freshwater fishes. Created in BioRender, (Schwieterman, G. (2025) https://BioRender.com/w27j369).

Conservation physiology offers an important approach for understanding and mitigating the threats facing freshwater ecosystems. Although several reviews have covered how conservation physiology can inform management and policy (Madliger *et al.*, 2016, Madliger *et al.*, 2020, Madliger *et al.*, 2018) as well as threat assessment and recovery planning of endangered species (Birnie-Gauvin *et al.*, 2017), those related to fishes have largely focused on marine ecosystems (*e.g.,*  Cooke *et al.*, 2014, McKenzie *et al.*, 2016) or a specific species (*e.g.,* Pacific salmon; Cooke *et al*., 2012). Our goal is to demonstrate how conservation physiology can improve the scientific knowledge base that underpins advice for the sustainable management of freshwater fishes experiencing a diversity of threats. We convened as a group of eleven experts in the field of freshwater fish conservation physiology (representing early to mid-career individuals employed in academic or government settings) to compile a list of important existing and emerging threats facing freshwater fishes. We acknowledge that our group of experts is limited to North America and Europe, which may have introduced bias into our perception of relevant stressors. All threats included here were mutually agreed upon. Here, we highlight these threats (Figure 1) and identify how they are being addressed using conservation physiology tools. We also discuss knowledge gaps surrounding multi-stressor impacts and approaches to addressing these and conclude with a discussion of the barriers and challenges associated with moving research to successful conservation management.

## Threats Being Addressed with Conservation Physiology

### Increased temperature

Temperature is considered the ecological master factor, governing the biochemistry, physiology, and behaviour of ectothermic fish (Brett, 1971). Specifically, acute warming temperatures are known to have a suite of physiological changes including but not limited to, increased ectotherm metabolism, mobilized glucose stores, induced changes in cell membrane composition, and upregulated some heat shock proteins (Eliason and Anttila, 2017, Fry, 1947). Chronic exposures to high temperatures can limit growth rates, increase susceptibility to disease, and even cause mortality (Alfonso *et al.*, 2020, Schulte *et al.*, 2011, Stewart *et al.*, 2015). Climate change and other anthropogenic effects are causing increased mean temperature, record-breaking heat waves, and greater thermal variability (Perkins-Kirkpatrick and Lewis, 2020, Van Vliet *et al.*, 2013). Freshwater fish are limited in their ability to move across large geographic areas, which reduces their capacity for behavioural thermoregulation and imposes physiological challenges. Conservation physiologists have successfully used several techniques to investigate temperature effects on freshwater fishes. Respirometry is the most common method used to determine metabolic demands and constraints on fish and can therefore help estimate thermal performances and tolerance under various thermal regimes (Norin and Speers-Roesch, 2020, Sinclair *et al.*, 2016). One frequently used measure of the metabolic constraints on fish is aerobic scope (AS), which is the difference between maximal oxygen consumption (maximum metabolic rate, MMR) and oxygen consumption at rest (routine or standard metabolic rate, SMR). Respirometry is commonly paired with other techniques including the critical thermal maximum (CT_max_) test (Desforges *et al.*, 2023), the Arrhenius breakpoint temperature (ABT) test (Casselman *et al.*, 2012, Gilbert and Farrell, 2021), transcriptomics (Mackey *et al.*, 2020, von Biela *et al.*, 2023), blood physiology (Handy *et al.*, 1999, Hasler *et al.*, 2017, Muusze *et al.*, 1998, Poluhowich and Parks, 1972, Winter *et al.*, 2018), muscle contractility (Woytanowski and Coughlin, 2013), behaviour (Brownscombe *et al.*, 2024, Zupa *et al.*, 2021), or sensory biology (Hasenei *et al.*, 2020) to reveal the temperatures at which fish are physiologically compromised. These metrics can be used to model thermal performance curves which help determine optimal temperatures for performance (T_opt_) across populations or species (Chen *et al.*, 2013, Claireaux *et al.*, 2013, Healy and Schulte, 2012). Additionally, these thermal thresholds can inform species abundance models to predict which species or populations may be most vulnerable to climate change (Wagner *et al.*, 2023).

One notable example of using respirometry to study the effects of increased temperature on fish comes from the Fraser River watershed in British Columbia, Canada, where summer water temperatures have increased by over 2°C since the 1950s (Patterson *et al.*, 2007) and are projected to continue increasing (Grant *et al.*, 2019). Alarmingly, high river temperature is correlated with elevated *en route* mortality for migrating adult Pacific salmon (Martins *et al.*, 2012). Researchers have demonstrated that Pacific salmon populations are physiologically adapted to their specific migration conditions (difficulty and thermal regime) (Crossin *et al.*, 2004, Eliason *et al.*, 2011, Lee *et al.*, 2003). For example, a comparison of thermal performance curves for aerobic scope (AS) across six populations of migrating adult sockeye salmon (*Oncorhynchus nerka*) demonstrated differences in the temperatures at which AS is optimal (T_opt_) and zero (T_crit_), with unusually high and broad thermal tolerance of the Chilko population. Researchers sought a mechanistic basis for these intraspecific differences, and through temperature-holding experiments revealed greater ventricular β-adrenoceptor density (*B*_max_) in the Chilko population compared to the other population with lower thermal tolerance (Eliason *et al.*, 2011). The Canadian Department of Fisheries and Oceans now uses population-specific thermal performance metrics and temperature-dependent mortality estimates to regulate flow release and inform harvest limits during heatwaves to best sustain fish populations (Hague and Patterson, 2014, Macdonald *et al.*, 2012, Patterson *et al.*, 2016).

### Eutrophication

Anthropogenic eutrophication is caused by nutrient enrichment (primarily nitrogen and phosphorus) from agriculture and urbanisation. Nutrient sources include point sources, such as wastewater treatment plants, and diffuse sources, such as agriculture and runoff. Natural freshwater systems are often phosphorous-limited but can be nitrogen- or co-nutrient limited (Conley *et al.,* 2009). Nitrates (NO_3_^-^) and nitrites (NO_2_^-^), ambient nitrates hereafter, as well as total ammonia (equilibrium between ionized ammonium NH_4_^+^ and ionized “free” ammonia NH_3_), are the most prevalent nitrogen pollutants that are both bioavailable to primary producers, and potentially harmful to fish (Edwards *et al.,* 2024; Rodgers, 2021). Excess nutrients trigger algal growth and subsequent decay, which causes increased turbidity, hypoxia, and changing pH; effects that are accelerated with increased temperature (Diaz and Breitburg, 2009; Rodgers, 2021). Nutrient enrichment is also associated with blooms of toxic cyanobacteria (O'Neil *et al.*, 2012), which can be harmful to fish. Therefore, freshwater fish in hypereutrophic systems must cope with sequential and possibly synergistic direct and indirect stressors from acute to chronic timescales.

Nitrogen pollutants lead to diverse and complex sublethal and lethal impairments in fish. Ambient nitrates enter the blood through the gills by facilitated diffusion in place of chloride (Cl^-^) ions (Williams and Eddy, 1986). High plasma nitrate levels can disrupt ion balances and oxidise hemoglobin into methemoglobin, a non-functional form that cannot bind oxygen (Jensen, 1987, Lefevre *et al.*, 2011). These lead to cardiorespiratory impairment. Specifically, a fish’s AS can decline with nitrate acclimation either due to decreasing MMR (Gomez Isaza *et al.*, 2020b, Gomez Isaza *et al.*, 2020c, Opinion *et al.*, 2020) or increasing SMR (Gomez Isaza *et al.*, 2020a). Additionally, nitrogen pollution can lead to decreased growth (NO_3_^-^, Monsees *et al*., 2017; NO_2_^-^, Kroupova *et al*., 2008), decreased swimming performance (Gomez Isaza *et al.*, 2020b), hyperkalemia (NO_2_^-^, Aggergaard and Jensen 2001), increased antioxidant activity (Presa *et al*., 2022), gill swelling and damage (NO_2_^-^, Kroupova *et al.*, 2008, NO_3_^-^, Monsees *et al*., 2017), enlarged liver (NO_3_^-^, Monsees *et al*., 2017), and enlarged spleen (NO_3_^-^, Opinion *et al*., 2020). Ultimately, nitrate exposure can be lethal to fish (*e.g.,* Gomez Isaza *et al*., 2021, Lefevre *et al*., 2011, Kroupova *et al*., 2008, Ferreira da Costa *et al*., 2004, Presa *et al*., 2022). Similarly, the effects of ammonia include, but not limited to, impaired neurological function and reduced ATP production, increased reactive oxygen species (ROS) and oxidative damage, reduced swimming capacity, lethargy and death (reviewed by Ip and Chew 2010, Randall and Tsui, 2002, Edwards *et al.,* 2024). Furthermore, the toxicity of total ammonia increases with increasing pH and temperature; theses effects are particularly concerning in changing climates.

Eutrophication is a widespread global issue with few management and remittance guidelines. For example, the Canadian Council of Ministers of the Environment (CCME) generated controversy with its freshwater nitrate guideline (for point and non-point sources) in 2003 (CCME, 2003), because it was based on the sensitivity of the Pacific treefrog (*Pseudacris regilla*), whereas many mines, which are point sources for nitrates, are situated at sites too cold for amphibians and do not discharge into amphibian habitat. By using physiological approaches to assess a broader range of species, the updated 2012 guidelines included toxicological studies of 12 species, including fish, amphibians, algae, and invertebrates (CCME, 2012). Recently, Health Canada modelled a species sensitivity distribution and used the 5th percentile to derive a guideline of 13 mg NO_3_^-^/L. The most sensitive species in the analysis was lake trout (*Salvelinus namaycush*; early life stages [embryo, larva, swim-up fry]), where swim-up fry had reduced growth in chronic development test after exposure to 28 mg NO_3_^-^/L (McGurk *et al.*, 2006). This updated guideline does not incorporate any toxicity modifying factors, such low chloride (Crawford and Allen, 1977, Jensen, 2003) and water hardness, or any of the indirect effects of eutrophication (such as hypoxia and decreases in pH). Of 13 Canadian provinces and territories, only five have adopted this guideline and only the province of Manitoba has regulations to enforce it. Total ammonia (TAN) guidelines for freshwater are issued more broadly, including by United States Environmental Protection Agency (US EPA 2013), by Australian and New Zealand Environment and Conservation Council and Agriculture and Resource Management Council of Australia and New Zealand (ANZECC and ARMCANZ 2000), and by Canadian Environmental Protection Act 1999; however not all consider secondary factors like temperature. Future work would benefit from approaching eutrophication as part of a multi-stressor framework, exploring the combined effects of nitrate exposure and other factors such as temperature and life-stage on lethal and sub-lethal outcomes.

### Habitat loss & fragmentation

Freshwater systems are a continuum of heterogeneous habitats that naturally rely on movement between systems. With the increasing human population and an ever-growing demand for space and resources, freshwater systems face fragmentation and isolation (Arthington *et al.*, 2016, Mota *et al.*, 2014). Nevertheless, unobstructed connectivity is essential to maintaining genetic flow (Coleman *et al.*, 2018) and access to various habitats for foraging or reproduction, which is particularly essential for diadromous species such as eels (*Anguilla* spp.), salmonids, shads (*Alosa* spp), and sturgeons (*Acipenser* spp.) (Arthington *et al.*, 2016). Additionally, human manipulation of waterways leads to more simplified, homogenous habitats that can alter or impair fish behaviour (*e.g.,* the removal of spawning substrates and three-dimensional structures that are important for behaviour, algal growth, and low-velocity zones) and environmental conditions (*e.g.,* temperature, oxygen, pollution) (Friberg *et al.*, 2016, Strailey and Suski, 2022).

One example of how physiological approaches can be used to assess the impacts of habitat fragmentation comes from a pressing issue in Europe: the disruption of migration passage of the endangered European eel (A. *anguilla* L.). Eels often must pass through dams, weirs, and hydropower plants. Researchers used acoustic and Passive Integrated Transponder (PIT) telemetry to assess mortality and return rates through different barriers and identified which barriers reduced escapement and caused migration delay (Piper *et al.*, 2013). To further understand physiological stress on those that do survive passage, Ammar *et al.* (2021) assessed damage, stress, and immune biomarkers in eels that successfully passed through turbines. They found that turbine passage caused internal and external damage, and altered plasma glucose levels, alternative complement (ACH50), lysozyme, and peroxidase activities, and total immunoglobulin content (Ammar *et al.*, 2021). These findings indicate that passage requires higher energy expenditure and disrupts immunity, highlighting the indirect consequences of passages and the importance of minimising such effects. These sub-lethal impacts may have serious consequences at the population level and are readily documented through the application of physiological methodologies.

### Invasive species

Aquatic invasive species pose a substantial threat to freshwater ecosystems as introduced or colonising species may alter community structure and habitat quality, and lead to significant economic consequences (Turbelin *et al.*, 2023). Freshwater communities particularly vulnerable to invasive species compared to others, such as terrestrial communities, because of the high frequency, duration, and magnitude of anthropogenic impacts on freshwater systems (Gherardi *et al.*, 2008; Moorhouse and Macdonald, 2015; Rahel, 2007). Anthropogenic alterations of waterways that facilitate the spread and establishment of invasive species include increasing watershed connectedness, habitat homogenization, and reducing native predators and competitors (see Moyle and Light, 1996; Scott and Helfman, 2001; Scott, 2006; Johnson *et al.*, 2008). Invasive species are regularly introduced into freshwater systems by intentional releases, ballast water, bait buckets, and boat trailers, to name a few. Invasive species have characteristics that give them a competitive advantage against native species and promote their spread (García-Berthou, 2007; Ricciardi and Atkinson, 2004). For example, one of the many competitive advantages successful invasive species often have over other species is a broad tolerance to environmental stressors (Komoroske *et al.*, 2020, Wellband and Heath, 2017). One strategy to evaluate invasion potential may include expanding physiological data on thermal limits with genomic studies of the invading population. Once established, invasive species impact native freshwater ecosystems via predation, competition, hybridization, disease transfer, and habitat modification (Gallardo *et al.*, 2016; Peeler *et al.*, 2011; Oliveira *et al.*, 2006). These can lead to devastating biological consequences, such as trophic cascades (Brett and Goldman, 1996; Brooks and Dodson, 1965) and extinctions of endemic species (Miller *et al.*, 1989).

A well-documented example of the impact of invasive species to freshwater systems is from the invasion of sea lamprey (*Petromyzon marinus*) to the Laurentian Great Lakes. Sea lamprey were able to colonise the upper Great Lakes after the construction of the Welland Canal and subsequently devastated the fishing industry in the region. Chemical pesticides have been used to dramatically reduce the sea lamprey population since the 1960s (Siefkes, 2017). Rotenone and antimycin are commonly used to remove invasive fish, however the compound 3-trifluoromethyl-4-nitrophenol (TFM) was found to be more toxic to lampreys than other species of fishes (Wilkie *et al.*, 2019). The compound niclosamide was then added to the TFM treatments to improve the efficiency of the sea lamprey control measures; however, niclosamide is less specific to lampreys. Through extensive physiological assessments, researchers have been able to determine the mechanism of toxicity in fishes of these compounds by interfering with mitochondrial adenosine triphosphate (ATP) production (*e.g.,*  Birceanu *et al.,* 2011, Borowiec *et al.,* 2022, Lawrence *et al.,* 2021a). Using RNA-sequencing approaches, researchers have been able to identify unique cellular responses to lampricide exposure that contribute to differences in TFM tolerance between sea lamprey and the most tolerant teleost species that has been tested, the bluegill (*Lepomis macrochirus*; Lawrence *et al*., 2021, 2023). Knowledge of physiological tolerances can also make the control of invasive species, like sea lamprey, more effective. For example, there is evidence that larval lamprey sensitivity to TFM is seasonal, peaking during late spring (Muhametsafina *et al.*, 2019, Scholefield *et al.*, 2008). Hlina *et al.* (2021) found that this seasonality is related to temperature, rather than energy stores or body condition. Thus, managers can choose to use TFM to control larval lamprey when the temperature is optimal. These studies demonstrate the importance of understanding how genomic and transcriptomic methods can complement traditional physiological assessments to determine the physiological responses to stressors and identify species-specific cellular responses to contaminant exposure.

### Changes in food availability & quality

Humans are altering the nutritional status of freshwater and anadromous fish by changing their food web structure and dynamics (*e.g.,* diet availability, options, and quality). These changes often co-occur or directly result from other anthropogenic stressors. Pollution, rising temperatures, harmful algal blooms, salinisation, and invasive species can all reduce food availability or alter food quality for endemic fish populations, while also impacting the animals’ ability to find, ingest, digest, and assimilate a meal (Birnie-Gauvin *et al.*, 2017, Eliason and Hardison, 2024, Reid *et al.*, 2019). In turn, changes in the fish’s nutritional status can negatively affect its immune function, environmental tolerances, growth, reproduction, inter and intra-specific behaviours, development, migratory timing, energy balance, and more (Archer *et al.*, 2019, Brett, 1971, Brett *et al.*, 1969, Chase *et al.*, 2016, Hansen *et al.*, 2020, Huey and Kingsolver, 2019, Mishra and Samantaray, 2004, Rodgers *et al.*, 2019, Turko *et al.*, 2020, Woiwode and Adelman, 1991). To manage food resources for freshwater fishes, researchers must (1) identify the cause of the nutritional stress, (2) determine the consequences and the severity of the nutritional stress on the animal, (3) evaluate remediation options, and (4) execute on an informed remediation plan. Conservation physiology can aid in determining the cause, consequences, and best course of action for correcting nutritional stress in freshwater fish.

For example, several salmonid species (*e.g.*, brown trout, Chinook salmon, coho salmon, steelhead trout, and more; Harder *et al.,* 2018) are vulnerable to a thiamine (vitamin B1) deficiency, which causes high mortality rates, neurological disfunction, and abnormal behaviour (*e.g.,* swimming, activity; Mantua *et al*., 2021; Harder *et al.,* 2018). In California, this has recently been observed in hatchery-reared Chinook salmon, where the deficiency has been linked to heightened anchovy consumption during the adult fish’s marine life stage, resulting from simultaneous reductions in other salmon prey and increases in anchovy abundance. Compared to other Chinook salmon prey, anchovy contain disproportionally high amounts of thiaminase, an enzyme that can degrade thiamine, which may explain why adult females returned from the ocean to freshwater with thiamine-deficient eggs (Harder *et al.*, 2018, Mantua *et al.*, 2021). The link between prey and thiamine deficiency was discovered through gut content analysis of adult Chinook salmon in their marine life stage and nutritional analysis of tissue from adults, eggs, and early life stages (Mantua *et al.*, 2021). Researchers have found that exposing fry to thiamine baths or directly injecting pre-spawn females with thiamine rescues the fish from the deficiency (Futia *et al.*, 2017). Notably, thiamine deficiency has been observed in several freshwater and anadromous fishes, and determining the exact cause of the deficiency and how to manage it in wild fish is an active area of research for conservation physiologists (Baker *et al.*, 2023, Harder *et al.*, 2018). For example, in the great lakes, the deficiency is similarly linked to thiaminase concentrations found in prey fish, like alewife (Harder *et al.,* 2018). While in a hatchery setting, thiamine deficiency can be mitigated through thiamine baths and other measures, managing this deficiency in wild fish populations is complicated. In lakes, successful mitigation of thiamine deficiency may be possible by managing prey populations to ensure sufficient availability of prey with low levels of thiaminase. However, implementing measures to mitigate thiamine deficiency is an ongoing challenge, especially in areas where conservation practices do not always align with community interests (Harder *et al.,* 2018). Despite these challenges, conservation physiology can help address nutritional stress by identifying deficiencies in wild fish stocks and informing potential solutions.

### Natural disasters

Natural disasters are extreme geophysical events that have profound effects on their surrounding environments. In freshwater systems, natural disasters include drought, wildfire, floods, landslides, and other extreme weather or geological events such as hurricanes and earthquakes (Cooke *et al.*, 2023). These events have occurred naturally throughout history and have shaped ecological processes and freshwater fish communities. Natural disasters impact freshwater fish populations through physical displacement, habitat loss, acute and chronic alterations in water quality, and changes in food availability and food web dynamics. For example, drought can lead to altered fish species assemblages due to the dominance of species with high thermal tolerance, generalist diets, and high dispersal abilities (Bond *et al.*, 2008, Chessman, 2013, Matthews and Marsh-Matthews, 2003). On the individual level, the environmental changes brought on by natural disasters can have physiological consequences that lead to fish mortality or reduced fitness. Landslides, for example, create energetic challenges for fish by mobilizing suspended sediments into waterways and by altering flow patterns (Cooke *et al.*, 2023). Excess suspended sediments can cause gill damage which, if nonlethal, has cascading effects on general stress levels and metabolic demands (Kemp *et al.,* 2011). High water velocities in narrowed stream or river channels where landslides have occurred increase the energetic effort required for migratory fish to pass through which can reduce the likelihood of migration success (Hinch *et al.*, 1996).

Natural disasters are becoming more frequent and often have more extreme effects on freshwater systems as a result of human-mediated impacts such as climate change, urbanisation, and water extraction. These changes can create challenges even for species that are adapted to withstand disturbances (Banholzer *et al.*, 2014, Bixby *et al.*, 2015, Bond *et al.*, 2008, Cooke *et al.*, 2023, Val and Wood, 2022). Different types of natural disasters can also increase the risk or exacerbate the effects of one another on freshwater fishes and their habitats. For example, dry vegetation resulting from drought leads to increased wildfire risk (Westerling *et al.*, 2006) and the destruction of vegetation by wildfire can increase the risk of landslides and flooding (Gomez Isaza *et al.*, 2022). Disasters that lead to habitat loss or reorganisation (*e.g.,* landslides, flooding, earthquakes) can make fish more vulnerable to disasters like drought where the availability of refuge habitats are crucial for fish persistence.

Physiological techniques can be used to assess the vulnerability of freshwater fishes to natural disasters. One example comes from prairie stream fishes' responses to drought. Hopper *et al.* (2020) found that differences in thermal tolerance between sympatric species of prairie stream fishes predicted their relative responses to severe habitat drying. A previously dominant species, southern redbelly dace (*Chrosomus erythrogaster*), was found to have the lowest thermal tolerance of the species tested, and field observations confirmed that this species experienced a 95% decline in abundance during the drought. The native central stoneroller (*Campostoma anomalum*) had a higher thermal tolerance and persisted across more pools than the southern redbelly dace. The invasive western mosquitofish (*Gambusia affinis*) had the highest thermal tolerance of these species and became the dominant species in the study-reach during the drought. This study is an example of how physiological tolerance data can be used to predict shifts in freshwater fish community structure during a natural disaster. Adaptive management practices that consider the physiology of freshwater fishes and that promote ecosystem resilience (*e.g.,* maintenance of natural flow regimes and native biodiversity; Lytle and Poff, 2004, Moberg & Galaz,, 2005, Orchard *et al*., 2017) are critical for mitigating the effects of natural disasters on freshwater fishes.

### Contaminants

The anthropogenic contamination of freshwater environments has exponentially increased in recent decades, making pollution in aquatic ecosystems one of the most significant contributors to biodiversity loss (Sigmund *et al*., 2023). Contaminants can be detected in most aquatic environments including isolated areas like the Arctic (Gauthier *et al.*, 2021) and have been shown to have a diverse range of impacts, with more than 11, 500 out of 83, 699 species considered to be impacted by pollution via the Red List led by the International Union for Conservation of Nature (IUCN, 2022). There is a diverse range of contaminants present in the environment; however they fall into one of two categories, the first being organic which are expansive and include chemicals like, pesticides, pharmaceuticals, perfluoroalkyl and polyfluoroalkyl substances (PFAS), polycyclic aromatic hydrocarbons (PAHs). The second contaminant class represents inorganics that include trace metals, metalloids, and some metal-based pesticides (Wood, 2012). Further, it is not uncommon to see mixtures of contaminant classes particularly during extreme weather events such as storm water runoff during heavy precipitation, or through industrial practices like hydraulic fracturing, all of which may cause a cascade of chemicals into the environment at once.

The issue of contaminants is particularly severe for fish in freshwater habitats, where exposure could occur via waterborne, dietary or sediment exposure and where factors such as their physiology and the physiochemistry of the water heighten their vulnerability to contaminants. Freshwater fish, being hyperosmotic to their surroundings, constantly lose ions across their body surface through diffusion. To counteract this, they must actively absorb ions against a concentration gradient, a process that critically depends on effective ion uptake from the environment (Evans *et al.,* 1999). Consequently, contaminants that disrupt ion regulation are particularly toxic to these fish (Wood, 2012). In the case of trace metals, this toxicity is exacerbated by freshwater chemistry, which limits complexation (the combination of atom groups, ions, or molecules to form larger molecules) and competition (two or more types of molecules can bind to the same site on another molecule), factors that reduce trace metal bioavailability, and thus toxicity (Di Toro *et al.,* 2009, Wood, 2012). There is a growing recognition of the importance of integrating physiological traits into conservation strategies to assess contamination-related risks within aquatic environments, helping to safeguard freshwater ecosystems.

A prime example of conservation physiology in action is the study of silver (Ag^+^) toxicity in freshwater organisms. Silver contamination in freshwater ecosystems originates from natural leaching, anthropogenic activities such as mining, and, historically, from photographic processing (Wood *et al.*, 2009). In the late 20th century, concerns emerged regarding silver used in photo processing, particularly because regulatory frameworks focused only on total metal concentration without considering metal speciation (e.g., Ag^+^ vs. AgCl) (Adams *et al.*, 2002). Laboratory-based toxicological assessments using model species such as Daphnia magna and rainbow trout (*Oncorhynchus mykiss*) revealed that these organisms were extremely sensitive to silver toxicity, with median lethal concentrations recorded in the low μg/L range. Further research demonstrated that only ionic silver (Ag^+^) is bioavailable and toxic, whereas other forms of silver, such as AgCl, are not. The physiological basis for this selectivity lies in the fact that ionic silver mimics the essential ion sodium (Na^+^), allowing it to be taken up by sodium transport pathways in the gill epithelium (Wood, 2012). In contrast, other silver species cannot utilise these pathways, preventing them from entering the organism, bioaccumulating, and causing toxicity. Any aquatic environmental variable, such as low pH, that promotes the formation of Ag^+^, will enhance silver toxicity to the organism, and would need to be accounted for in toxicity studies. Recognizing the physiological basis for Ag^+^ toxicity has led to the development of site-specific water chemistry models and geochemical tools used by regulatory bodies to predict metal toxicity (Glover, 2018).

### Noise & light pollution

Anthropogenic noise and light pollution are pervasive stressors for freshwater fish, as they rely on sound and vision for cues coordinating reproduction, detecting predators, and identifying habitat (Longcore and Rich, 2004, Mickle and Higgs, 2018, Perkin *et al.*, 2011, van der Sluijs *et al.*, 2010). Since many freshwater ecosystems are located near urban developments, noise and light pollution threaten the reception of the sensory information that fishes rely upon. There is evidence that noise increases glucocorticoid production (Crovo *et al.*, 2015, Wysocki *et al.*, 2006) and cardiac output (Graham and Cooke, 2008) in freshwater fish and can induce hearing loss (Crovo *et al.*, 2015, Smith *et al.*, 2004). A recent meta-analysis of 42 fish studies (not freshwater specific) revealed that, generally, anthropogenic noise increased movement and reproduction related behaviours, decreased foraging behaviors, increased the hearing threshold (animals had more difficulty hearing), and increased physiological indicators of stress (Cox *et al.*, 2018). Characterising the impacts of light and noise pollution on fish physiology and behaviour allows managers to mitigate these threats. Light pollution is well-documented to have effects on organisms, including freshwater fish (e.g. Foster *et al.,* 2016; Tarena *et al.*, 2024; Vowles and Kemp, 2021), leading to several initiatives aimed at reducing artificial light at night (ALAN) around the globe (Clavier, 2024). Light underlies biological rhythms via changes in diel, lunar and seasonal cycles, and organisms are tuned to these periodic light changes via their circadian rhythms to balance physiological functions and behaviour to time of day, lunar phase and time of year. Circadian rhythms play a particularly important role in the repair and recovery of physiological functions during periods of rest or dormancy (Aronson *et al.*, 1993). Studies have shown that disruptions of circadian rhythms can lead to a reorganisation of the entire physiological state of an organism, including the suppression of melatonin (Brüning *et al.*, 2015). For example, light pollution found on docks and shoreline habitats, even intermittent sources, can increase activity levels in fish, potentially impacting energy expenditure and behaviours such as nest-guarding by smallmouth bass (*Micropterus dolomieu*) (Foster *et al.*, 2016). Given that urban development is rampant, noise and light pollution will continue to threaten the well-being of fish in freshwater ecosystems (Hölker *et al.*, 2023).

Reproductive periods are particularly challenging for many species of fish (Dahlke *et al.*, 2020) and may represent a time of heightened vulnerability to the effects of noise (Blom *et al.*, 2019, de Jong *et al.*, 2018, de Jong *et al.*, 2020, Nedelec *et al.*, 2017, Sierra-Flores *et al.*, 2015). To study the effects of noise on maternal care and brood development, Butler and Maruska (2021) exposed mouth-brooding African cichlid (*Astatotilapia burtoni*) females to 3 h of noise (140 dB) half-way through their brooding period. Fifty-five percent of the females exposed to noise cannibalised or released their young prematurely, compared to 10% of the control females. Using RNA-seq analysis, the authors found that transcripts related to feeding and brood care were differentially expressed in the group treated with noise. Furthermore, juvenile fish from broods exposed to noise had lower body condition, altered head transcriptomes, delayed onset of adult colouration and behaviour, and higher mortality compared to those from the control treatment broods. Conservation physiology studies have clearly demonstrated disruptions to the physiology and ecology of fishes exposed to these threats, underscoring the need to reduce them. We are not aware of examples of studies that have specifically influenced management related to light and noise pollution in freshwater fishes, but such examples do exist within the marine and terrestrial realms. These include the “Lighting the right place, at the right time, when it is needed” approach in France for example (Lapostolle and Challéat, 2020). A similar approach could be used in freshwater (Harrison and Gray, 2024, Pérez Vega *et al.*, 2024).

### Infectious diseases

Fish disease in freshwater systems can be detectable from a physiological perspective as a decrease in a host’s ability to meet basic fitness demands (*e.g.,* feed, reproduce, avoid predation). Pathogens come in many forms (viruses, bacteria, parasites, etc.) and are ubiquitous in freshwater environments, but only cause disease under conditions which compromise both the host and environment. Extreme infectious disease outbreaks may be visible as mass die-offs, but less dramatic impacts can also affect the productivity and resilience of freshwater fish populations, especially in the context of cumulative stressors related to climate change and urbanisation (Lane *et al.*, 2020). Physiological tools can be used to detect changes that indicate disease *potential* and, when combined with shifts in pathogen loads, tell a comprehensive story of wild fish health that can inform management. These tools may be applied at single or multiple levels of biological organisation to identify markers at genomic (*e.g.,* immune gene regulation, pathogen genetic material in host, eDNA), cellular (*e.g.,* immune cell migration, histology), organismal (*e.g.,* organ function, energetics), or para-organismal levels (*e.g.,* mucosal microbiota) and can also be paired with behavioural assessments (*e.g.,* observation, telemetry).

Physiological research can reveal how fish immune responses and disease potential respond to changes in environmental conditions. Temperature can modulate the type of immune response a host will elicit; for example, a study of perch (*Perca fluviatilis*) found that specific recognition (adaptive immunity) was higher in fish reared at elevated temperatures (Marnila and Lilius, 2015). However, decreased temperatures can also slow physiological responses, including the recognition of and defence against pathogens. Chandra *et al.* (2023) found that the reaction of lymphocytes (innate immune response) from the anterior kidney and spleen of male snakeheads (*Channa punctatus*) had a three-month rhythm in their reaction to three different mitogens and that these rhythms peaked in the same months. They also observed a slowing of the immune response during winter conditions when the energetic cost of immunity is relatively high due to lower nutrient intake during these months. Predicting and addressing emerging threats to freshwater fishes can be achieved by linking energetic capacity and immunity, thereby considering each animal as a *system* in which pathogens can thrive or perish. Understanding how fish immune responses are adapted to seasonality can inform responses to disease in fish, especially as environmental conditions change in response to climate change.

## Considering the Human Dimension in Freshwater Fish Conservation Physiology

One of the hurdles for freshwater conservation physiology, indeed for all of science, is the historical restriction of participation in the scientific and conservation processes to those with the access and means to obtain training in western science methodologies. Despite studies demonstrating the positive impact of involving people of diverse identities in research efforts (Birnie-Gauvin *et al.*, 2023; Fehr, 2011; Intemann, 2009; Smith-Doerr *et al.*, 2017), many identities have been excluded from participating in the fields of conservation physiology and fisheries science through lack of opportunity, passive inaction, bias, discrimination, and active exclusion (e.g., persons of color, LGBTQIA+ individuals, female-identifying persons, those with low socioeconomic status, etc.) (Bernard and Cooperdock, 2018, Li and Koedel, 2017). The slow pace with which the field has addressed these harmful practices has held back the advancement of conservation physiology. For example, by not valuing the voices and perspectives of indigenous peoples and local communities, scientists have limited their ability to ask relevant or informed questions about freshwater species and systems. By working with local communities and valuing traditional knowledge systems, we can build collective understandings that benefit both research and traditional land and water stewards (Naskar *et al.*, 2017, Reid *et al.*, 2022). For example, freshwater conservation physiology was leveraged to help win a British Columbia Supreme Court case Thomas and Saik’us First Nation v. Rio Tinto Alcan Inc (2022) by illustrating the negative impact of warm temperature on migrating pacific salmon populations (Eliason *et al.,* 2011). Engaging with a more diverse array of individuals can also help ensure that work is more meaningfully translated into action. While studying physiology can help to inform conservation actions, measurable success in conservation is nearly always a partnership across sectors to balance the needs and desires of different user groups.

At the other end of the knowledge pipeline, many physiologists also struggle to meaningfully engage with politicians and policy makers, perhaps a result of comparative animal physiology’s historic relegation to academic settings. Collaborating with policy makers from the onset of a project ensures that the final products are both practical and effectively distributed to end users. This maximises the impact of the labour and funding invested in data collection and interpretation (Cooke and O’Connor, 2010, Rose *et al.*, 2020).

## Approaches to Addressing Conservation Physiology Problems on the Horizon

Here, we have illustrated a suite of conservation issues facing freshwater fishes and highlighted examples of how physiological tools can help shed light on pathways towards effective management. However, the real world is often complex in nature and conservation issues rarely occur in isolation (Birk *et al.*, 2020, Jackson *et al.*, 2016, Ormerod *et al.*, 2010). While others have detailed the current state of multi-stressor studies in freshwater systems, limitations in fully factorial designs and a lack of mechanistic understandings driving observed biological responses to stressors limit our understanding of these potentially interactive threats (see Jackson *et al.*, 2016, Orr *et al.*, 2024). For example, eutrophication increases under warming temperatures and causes changes in oxygen levels as well as in nitrites. Similarly, rising temperatures can increase susceptibility to infectious diseases, potentially increasing the invasion potential for pathogen-resistant species. Multiple concurrent stressors are not only extremely common, but are also generally understudied due to the increased cost and inherent difficulty associated with determining causal relationships (Lima *et al.*, 2023). Whether simultaneous application of threats has additive, synergistic, or antagonistic impacts is situationally dependent (Orr *et al.*, 2024), although a recent meta-analysis has shown a high prevalence of masking effects in multi-stressor scenarios (Morris *et al.*, 2022). The authors feel that conservation physiology is uniquely positioned to tease apart the impacts of multi-stressor experiments by emphasizing causal mechanisms rather than relying upon correlative observations. This mechanistic perspective has been shown to improve habitat modelling using single stressors (e.g., temperature; Patterson *et al.,* 2016), and a similar approach holds promise for multiple concurrent stressors (e.g., hypoxia and temperature; Arend *et al.,* 2011). A physiological approach still must reconcile the logistical, financial, and methodological challenges of factorial experiments, as well as the challenges of characterizing threshold effects, and time courses of exposure to threats. However, by emphasizing process at the individual and population level, we feel physiology is one of the more promising paths towards effective conservation measures.

**Figure 2:**
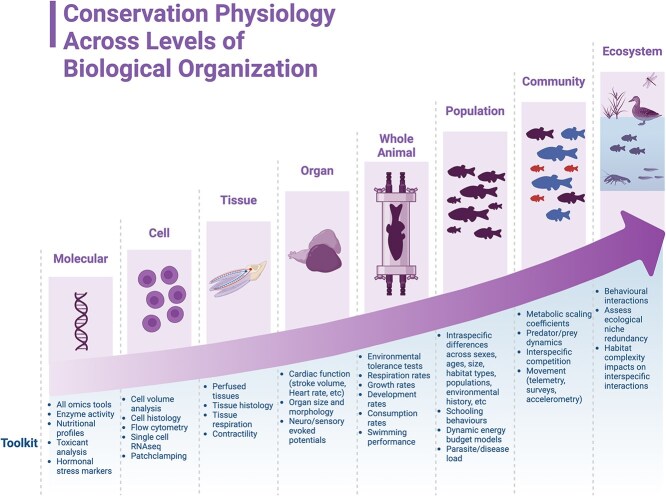
Physiological tools that can be used across levels of biological organisation to inform freshwater conservation. Created in BioRender, (Hardison, E. (2025) https://BioRender.com/g65t345).

It is an urgent challenge for the field of freshwater fish conservation physiology to reconcile the mechanistic understandings with management actions at broad spatial and temporal scales (see also Harper *et al*., 2021). Here, the authors summarize what we feel to be some important guiding questions that help researchers design conservation-minded physiological experiments with high impact: (*1*) How does the intensity, frequency, and duration of the threat impact physiological responses, and how does this vary with species, life stage, nutritional state, or other co-stressors? (*2*) How do standard physiological metrics (*e.g.,* AS, CT_max,_ blood cortisol) translate to behavioural or other sub-lethal changes which can impact overall fitness, and what additional biomarkers can be identified that are predictive of performance/fate? (*3*) What are the epigenetic impacts of stress, and how can these be mitigated? (*4*) What has driven past conservation successes and failures, and can these lessons be scaled/adapted to different environments? (*5*) How can freshwater scientists work more closely with community partners (including but not limited to indigenous peoples, recreational users, fishers, and policy makers) throughout the research process to improve transparency, knowledge sharing, and the efficacy of management plans relative to socio-economic and cultural contexts?

## Conclusions

Freshwater fishes are facing serious conservation threats. Here, we have illustrated how various physiological tools can inform freshwater fish management in the face of numerous stressors (applied both in isolation and concurrently) and highlighted some exciting new developments that will continue to grow the applicability of physiological data in freshwater conservation (Figure 2). Despite the challenges in linking physiology to application (Cooke and O’Connor, 2010), we believe that physiology is essential in the creation of effective conservation strategies. Physiology has the capacity to explain how individuals interact with their habitats and ecosystems, thus driving life’s processes including behaviour, fitness, and mortality. By leveraging the wealth of physiological tools at our disposal, we have an opportunity to increase the efficacy of conservation policies in freshwater systems and improve our ability to build sustainable communities for years to come.

## Data Availability

This is a synthesis article so there are no data or code to share.
